# Natural Killer Cell-Mediated Shedding of ULBP2

**DOI:** 10.1371/journal.pone.0091133

**Published:** 2014-03-10

**Authors:** Ruipeng Wang, Peter D. Sun

**Affiliations:** Structural Immunology Section, Laboratory of Immunogenetics, National Institute of Allergy and Infectious Diseases, National Institutes of Health, Rockville, Maryland, United States of America; INSERM- CNRS- Univ. Méditerranée, France

## Abstract

UL16 binding proteins (ULBPs) are a family of cell surface proteins that are present in transformed and stressed cells and ligands for NKG2D. Soluble NKG2D ligands have been found in sera from cancer patients with their protein concentrations correlated with poor cancer prognosis. Here we show, for the first time, that human tumor cells lost their surface expression of ULBP2, but not ULBP1 and ULBP3, during NK cell-mediated cytolysis. In contrast to spontaneous shedding of NKG2D ligands, NK cytolysis-mediated shedding of ULBP2 was linked to target cell apoptosis, although both resulted from metalloproteinase cleavages. Inhibition of ULBP2 shedding by a metalloproteinase inhibitor BB-94 lead to reduced NK cell-mediated cytotoxicity and cytokine production. These results illustrate a regulation of NK cell effector functions through cytolysis-induced NKG2D ligand shedding. Consequently, compounds inhibiting NKG2D ligand shedding may offer therapeutic means to reduce excessive pathogenic NK cell activities.

## Introduction

UL16 binding proteins (ULBPs) are a family of cell membrane proteins expressed on both transformed and stressed cells. They were identified by their ability to bind to human cytomegalovirus protein UL16 [1]. In humans, ULBP family proteins contain 6 members, including GPI anchored proteins ULBP1-3 and 6, and transmembrane proteins ULBP4-5 [2], [3]. ULBPs, as well as MHC class I-related chain (MIC) A and B proteins, are ligands of NKG2D [4]–[6], an activating receptor expressed on natural killer (NK) cells, CD8 T cells, γδ T cells and some CD4 T cells [7]. Ectopic expression of mouse NKG2D ligands on tumor cells promotes NK cell recognition and enhances tumor rejection in syngeneic mice [8]. In spontaneous cancer models, NKG2D deficiency gives rise to a higher incidence of malignancies in mice [9].

Soluble NKG2D ligands can be released by tumors, which have been identified as markers for tumor prognosis. For example, the concentration of ULBP2 in serum appears to be associated with poor survival in melanoma, B-cell chronic lymphocytic leukemia and lung cancer patients, and therefore it can be a marker for tumor load [10]–[13]. Considering the important role of NKG2D ligands in shaping NKG2D-related effector functions [2], [3], it is interesting to understand how the NK cell effector functions, including cytotoxicity and cytokine production, could affect the expression of NKG2D ligands. In this study, we show a specific shedding of cell surface ULBP2 induced by NK cell-mediated cytolysis, which is more intense and faster than previously reported spontaneous shedding of it. The same as spontaneous shedding, NK cell/apoptosis-induced shedding of ULBP2 also requires metalloproteinases, since it can be abrogated by broad spectrum metalloproteinase inhibitor BB-94. Interestingly, block shedding of ULBP2 by adding BB-94 reduced NK cell-mediated cytolytic function and IFN-γ production. Together, our results show faster NK cell-induced shedding of ULBP2 which also contributed to modulation of NK cell effector functions.

## Materials and Methods

### Cells, Cytokines and Reagents

Human NK cells were isolated from peripheral blood lymphocytes of unidentified donors (NIH blood bank) by negative selection using the EasySep™ human NK cell enrichment kit (STEMCELL Technologies). Purified NK cells were co-cultured with an equal number of Mitomycin C (Roche Diagnostics)-treated autologous PBL feeder cells in IMDM (Life Technologies) supplemented with 10% human AB serum (Sigma-Aldrich), 10% purified IL-2 (Hemagen Diagnostics), 200 U/ml recombinant human IL-2 for one week, and then expanded NK cells were cultured with IMDM supplemented with 10% human AB serum and rIL-2 (200 U/ml). All cell lines were from the American Type Culture Collection (Manassas, VA, USA). Recombinant human IL-2 was from the National Cancer Institute-Frederick Cancer Research and Development Center (Frederick, MD, USA). Actinomycin D (ActD), Camptothecin (CPT) and Etoposide (ETO) were from Sigma-Aldrich. Z-VAD-FMK and Z-FA-FMK were from BD Biosciences. The synthetic metalloproteinase inhibitor BB-94 (Batimastat) was from Santa Cruz Biotechnology.

### Apoptosis Assays

For treatment with ActD, CPT and ETO, Jurkat or H9 cells were cultured in serum-free RPMI 1640 medium with the indicated amount of chemical apoptosis inducer. To block the apoptosis induced by these chemicals, 50 µM Z-VAD-FMK was used to pre-treat Jurkat cells at 37°C for 30 min, and 50 µM Z-FA-FMK and DMSO were used as controls. For heat shock treatment, Jurkat cells were resuspended in serum-free RPMI 1640 medium and heat-shocked at 45°C for 30 min. The heat shocked cells were divided into two aliquots; one was cultured at 37°C for 2 hours to induce apoptosis, and the other used as a control was placed on ice until it was subjected to flow cytometric analysis. To block the shedding of ULBP2, 5 µM BB-94 was added into cell cultures along with apoptosis inducers or NK cells simultaneously.

### Flow Cytometric Analysis

Cells used for flow cytometric analysis were pre-incubated with human IgG (10 µg/ml; I4506; Sigma) on ice for 20 min. For flow cytometry staining, the following antibodies were used: FITC/PE/allophycocyanin (APC)-conjugated anti-human CD56 (B159; BD Pharmingen), PE-conjugated anti-human MICA/B (6D4; BD Pharmingen), PE-conjugated anti-human CD107a (H4A3; BD Pharmingen), PE-conjugated anti-human ULBP1 (170818; R&D Systems), and PE-conjugated anti-human ULBP2 (165903; R&D Systems). Alternatively, cells were incubated with biotinylated goat anti-human ULBP1 and ULBP2 polyclonal antibodies (R&D Systems) or unconjugated monoclonal antibodies against ULBP1, ULBP2, ICAM1 (HCD54; BioLegend) and ICAM2 (86911; R&D Systems), followed by APC-conjugated streptavidin or APC-conjugated anti-mouse IgG secondary antibody staining. After washing cells with PBS (containing 2% FBS), the labeled cells were analyzed by FACSCalibur (BD Biosciences). Small cellular debris and clumps were gated out of the data analysis. FlowJo software (Tree Star) was used for data analysis.

### Confocal Microscopy

Jurkat cells were treated with 4 µg/ml Actinomycin D at 37°C for 2 hours in serum-free RPMI 1640 medium in the presence or absence of BB-94 (5 µM). The treated cells were stained by mouse anti-human ULBP2 mAb (165903; R&D Systems), followed by DyLight 594 goat anti-mouse IgG (minimal x-reactivity) antibody (BioLegend) staining. After washing with cold PBS, cells were resuspended in Annexin V binding buffer and stained with Annexin V-FITC (BD Biosciences). The stained cells were relocated into Lab-Tek™ II chambered coverglass (155382, Thermo Scientific) and diluted 5 times by Annexin V binding buffer. Confocal images were acquired using a Zeiss LSM710 microscope with a Plan-Apochromat 63×1.40 oil DIC MC27 objective and ZEN2010 software. Scale bar, 10 µm.

### ELISA

Culture medium supernatants from Jurkat and H9 cells with or without treatments were collected at the indicated time points. The concentration of ULBP2 in the supernatant was analyzed by a human ULBP-2 DuoSet ELISA kit (R&D Systems), according to the manufacturer’s instructions. For IFN-γ detection, an IFN-γ DuoSet ELISA kit (R&D Systems) was used, and assays were carried out following the manufacturer’s instructions.

### Exosome Enrichment and Detection

2×10^7^ H9 cells were resuspended in 10 ml serum-free RPMI 1640 medium and treated with ActD or CPT overnight. Exosomes in H9 cell culture supernatants were enriched by using a Total Exosome Isolation (from cell culture media) kit (Life Technologies), according to the manufacturer’s instructions. Flow cytometry analysis of exosomes was performed using a previously published protocol with some minor modifications [14]. Briefly, 200 µl exosome preparations, prepared from 2 ml H9 culture supernatant, were incubated with 2 µl of 4-µm-diameter aldehyde/sulfate latex beads (Life Technologies) for 4 hours at room temperature and washed 3 times with PBS containing 2% FBS. Exosome-coated beads were resuspended in 200 µl PBS containing 2% FBS, and 100 µl resuspended beads were incubated with the following antibodies: PE-conjugated anti-human CD63 (H5C6; BD Pharmingen), PE-conjugated anti-human ULBP2 (R&D Systems) for 30 minutes at room temperature, the beads were washed and analyzed by a FACSCalibur flow cytometer (BD Biosciences).

## Results

### NK Cell Cytotoxicity Leads to Selective Loss of NKG2D Ligand on Killed Target Cells

**Figure 1:**
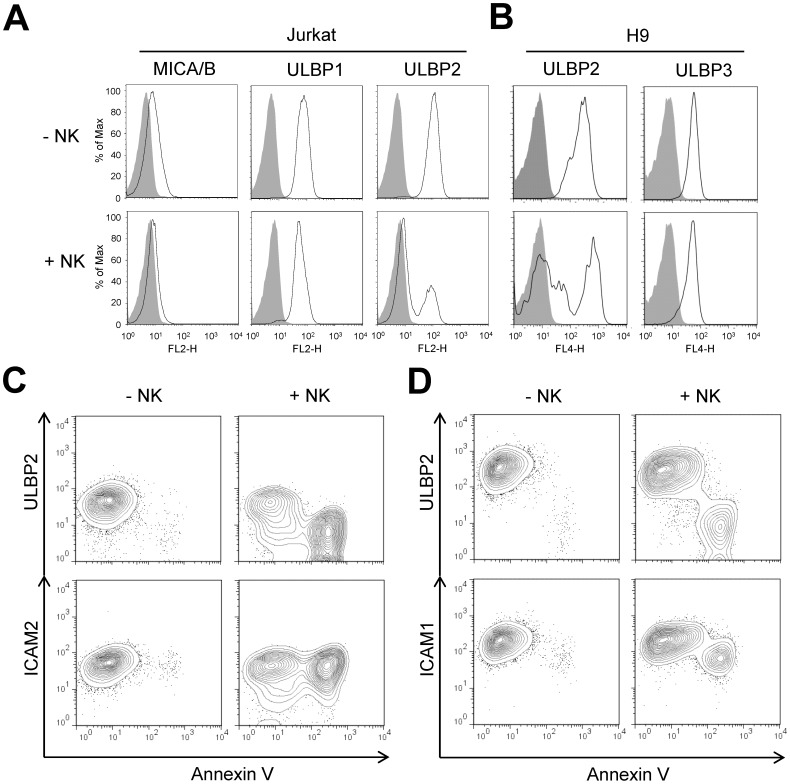
Loss of ULBP2 on target cells during NK cell-mediated cytolysis. (A, B) NK cell-mediated specific down-regulation of ULBP2. 10^5^ Jurkat (left panels) or H9 cells (right panels) were incubated with (+NK) or without (−NK) in an equal number of IL-2 expanded peripheral blood NK cells at 37°C for 2 hours. The resulting cell mixtures were stained by anti-human MICA/B, ULBP1, ULBP2 or ULBP3 antibodies and analyzed by flow cytometry (solid lines). NK cells were excluded by anti-human CD56 mAb staining. Isotype controls are shown in gray-shaded histograms. (C, D) NK cell-mediated target cell apoptosis leads to loss of ULBP2. 10^5^ Jurkat (C) or H9 cells (D) were incubated with (+NK) or without (−NK) in an equal number of IL-2 expanded peripheral blood NK cells at 37°C for 2 hours. The resulting cell mixtures were stained by anti-human ULBP2 or ICAM1/2 antibodies, followed by APC-conjugated goat anti-mouse IgG antibody and Annexin V-PE staining, and then analyzed by flow cytometry. NK cells were excluded by FITC conjugated anti-human CD56 mAb staining.

Target cell recognitions activate NK cells to secrete IFN-γ and TNF-α, which potentiates NK cell-mediated cytolysis of target cells through up-regulating ICAM-mediated NK-target adhesion instead of activating receptor ligands [15]. Interestingly, the presence of NK cells significantly reduced the expression of ULBP2, but not ULBP1 or ULBP3, on Jurkat and H9 cells (Fig. 1A and B). In addition, the percentage of ULBP2 low-target cells was closely correlated with effector-to-target ratios (E:T ratios) (Fig. S1), indicating that the loss of cell surface ULBP2 is associated with NK cell-mediated cytolysis of target cells. The bimodal distribution of target ULBP2 expression during NK cell-mediated cytolysis suggested the ligand was selectively lost only on some target cells rather than uniformly down-regulated on every target cell. Indeed, ULBP2 expression was lost only on killed target cells but remained unchanged on live target cells (Fig. 1C and D). The loss of NKG2D ligand expression on killed target cells, however, was selective and not a result of overall loss of cell surface markers on dead cells, because the expression of ULBP1 and adhesion molecules ICAM2 had not been affected, and the expression of ICAM1 only showed modest reduction.

### Apoptosis Results in the Loss of ULBP2 Expression

**Figure 2:**
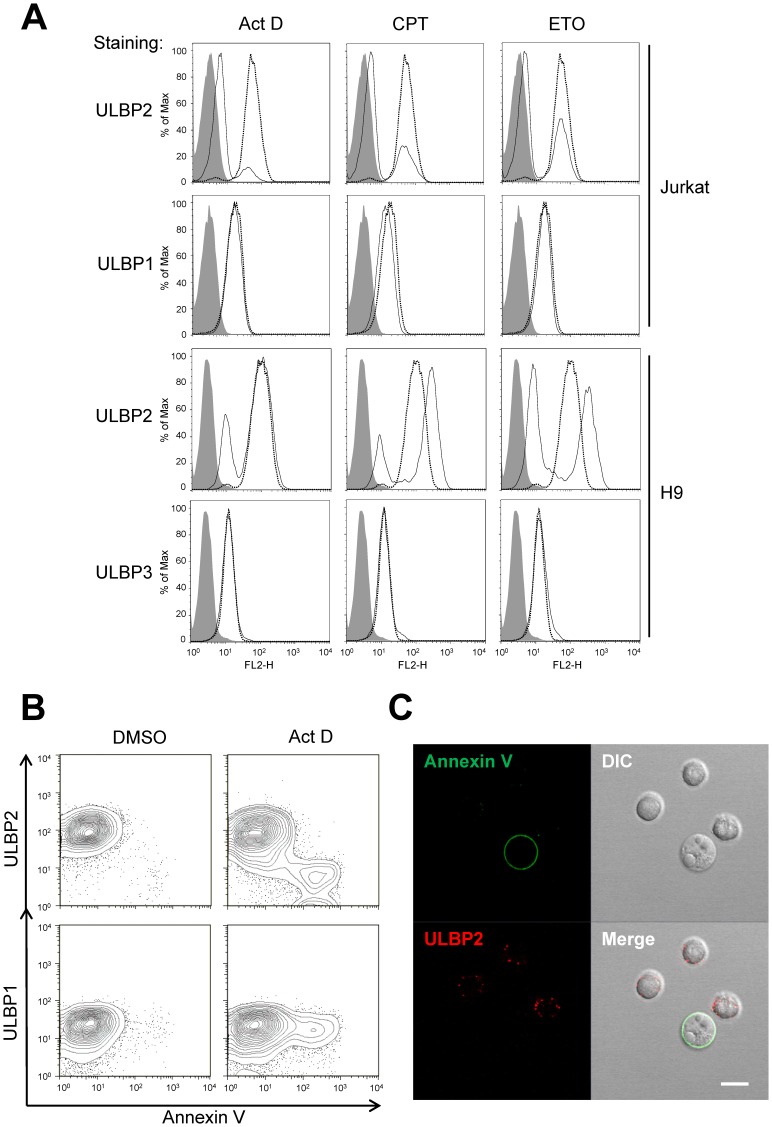
Apoptotic compound treatment also leads to loss of cell surface ULBP2. (A) Loss of cell surface ULBP2 expression in apoptotic compounds-treated cells. Jurkat cells (upper panels) were treated with 4 µg/ml Actinomycin D (ActD), 4 µM CPT, 25 µM ETO or DMSO for 4 hours in serum-free RPMI 1640 medium, and then were collected for flow cytometry staining. PE-conjugated mouse anti-human ULBP1 and ULBP2 antibodies were used. H9 cells (lower panels) were treated with 4 µg/ml ActD, 4 µM CPT or 50 µM ETO for 12 hours in serum-free RPMI 1640. DMSO-treated cells were used as the control (dotted lines). Biotin-labeled goat anti-human ULBP2 and ULBP3 and PE-conjugated streptavidin were used in this experiment. ULBP1/2/3 expression on control cells and treated cells are shown in dotted lines and solid lines, respectively. Isotype controls are shown in gray-shaded histograms. (B, C) Absence of ULBP2 on Annexin V positive cells. (B) Jurkat cells were treated with ActD for 2 hours in serum-free RPMI 1640 medium. The treated cells were stained by biotin-labeled goat anti-human ULBP1 or ULBP2 polyclonal antibodies, followed by APC-conjugated streptavidin and Annexin V-FITC staining, and then analyzed by flow cytometry. (C) Jurkat cells were treated with ActD for 2 hours, and then ULBP2 and Annexin V staining was visualized by confocal microscopy. Scale bar, 10 µm.

Since NK cell-mediated cytolysis involves perforin/granzyme- and/or Fas ligand-induced target cell apoptosis [6], and the loss of ULBP2 occurred only on killed target cells, we then examined if apoptosis accounts for the near complete loss of cell surface expression ULBP2. Actinomycin D (ActD), Camptothecin (CPT) and Etoposide (ETO) are known compounds to induce apoptosis [16], [17]. Jurkat cells treated with these apoptosis inducers lost their ULBP2 expression in 4 hours (Fig. 2A, upper panels). The same treatments did not affect the expressions of ULBP1, CD95 and class I MHC molecules (Figs. 2A and S2). The loss of ULBP2 expression was also detected on H9 cells treated with ActD, CPT or ETO for 12 hours (Fig. 2A). Similar to NK cell-mediated loss of ULBP2, the apoptotic compounds resulted in selective loss of ULBP2 but not ULBP1, ULBP3 and other cell surface markers, and the expression of ULBP2 became bimodal distribution. Indeed, the loss of ULBP2 expression was specific to Annexin V positive apoptotic cells in ActD-treated Jurkat cells (Fig. 2B). In contrast, ULBP1 expression remained unchanged in both apoptotic and non-apoptotic cells (Fig. 2B, lower panels). The similar results were obtained from heat-induced apoptosis of Jurkat cells (Fig. S3). The absence of ULBP2 in apoptotic cells is also evident in confocal images of ActD-treated and Annexin V-stained Jurkat cells (Fig. 2C).

### NK Cell-mediated Loss of ULBP2 Occurs through Apoptotic Pathway

**Figure 3:**
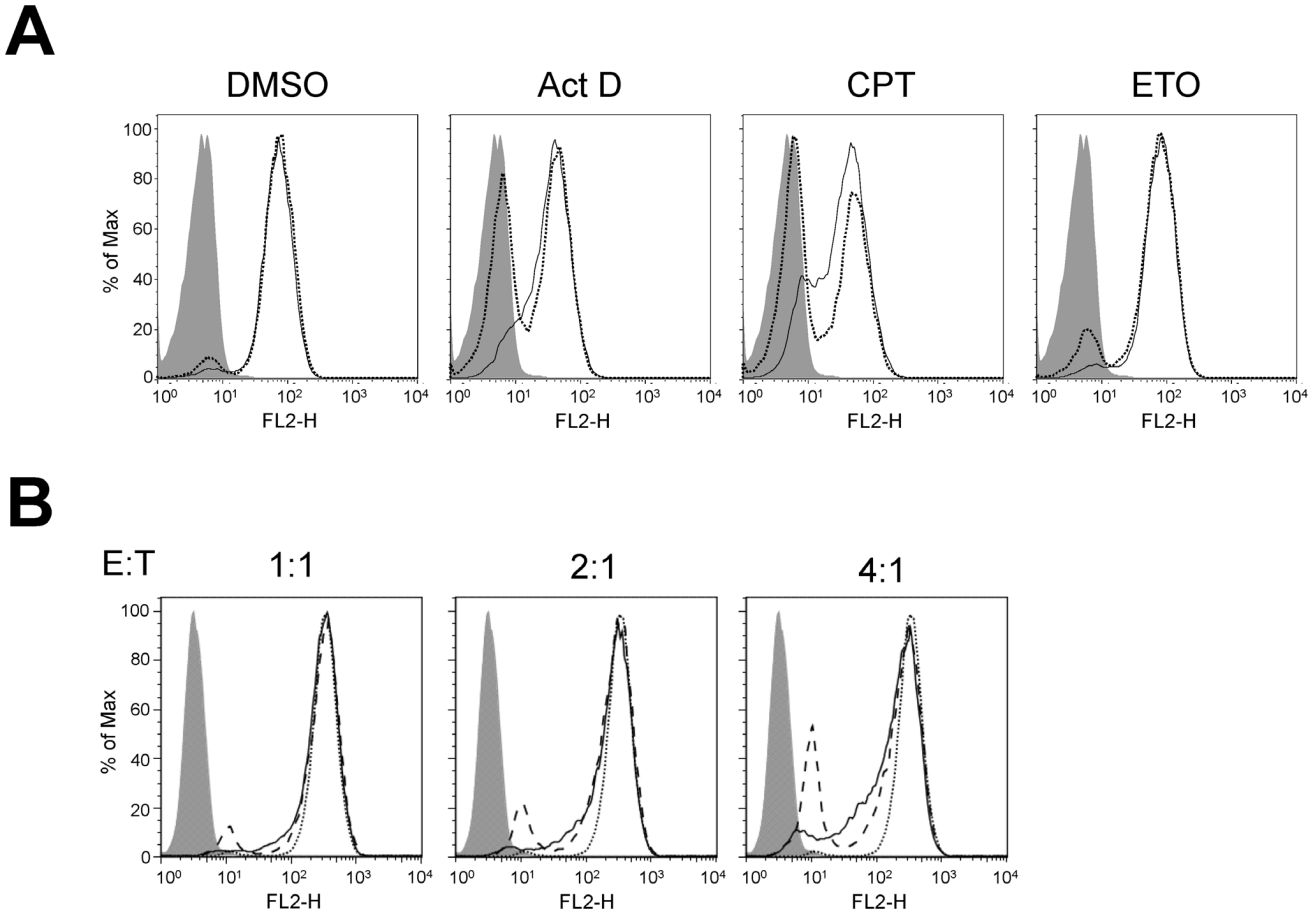
Inhibition of ULBP2 loss by apoptotic inhibitor. (A) Z-VAD-FMK inhibited apoptotic compound-induced loss of cell surface ULBP2 in Jurkat cells. Jurkat cells were pretreated with 50 µM Z-FA-FMK (dotted lines) or Z-VAD-FMK (solid lines) for 30 min, and then were treated with 2 µg/ml ActD, 4 µM CPT or 20 µM ETO for 4 hours at 37°C. Cell surface expression of ULBP2 was determined by flow cytometric analysis using PE-conjugated mouse anti-human ULBP2. PE-conjugated mouse IgG2a was used as an isotype control (gray-shaded). (B) Z-VAD-FMK inhibited NK cell-mediated loss of cell surface ULBP2 in H9 cells. 1.5×10^5^ H9 cells were incubated with IL-2 expanded human primary NK cells at the indicated E:T ratios for 2 hours, and the cell surface expression of ULBP2 was determined by flow cytometric analysis using PE-conjugated mouse anti-human ULBP2 antibody. NK cells were gated out by FSC/SSC and APC-conjugated CD56 antibody. ULBP2 expression in control H9 cells in the absence of NK cells are shown in dotted lines, whereas ULBP2 expression in H9 cells incubated with NK cells are shown in dashed lines (DMSO) and solid lines (Z-VAD-FMK), respectively.

Both NK cell- and apoptotic compound-induced loss of ULBP2 expression on target cells suggests that the loss of ULBP2 during NK cell-mediated cytolysis is linked to target cell apoptotic pathways. To further confirm this, we used a pan-caspase inhibitor Z-VAD-FMK (VAD) to inhibit the apoptosis-related down-regulation of ULBP2 in Jurkat cells. As expected, VAD, but not its control Z-FA-FMK (FA), inhibited ActD-, CPT- and ETO-induced loss of ULBP2 expression (Fig. 3A). Similarly, NK cell-mediated cytolysis induced loss of ULBP2 expression in H9 cells can also be inhibited by Z-VAD-FMK (Fig. 3B). Thus, the loss of NKG2D ligand expression during NK cell-mediated cytolysis is linked to target cell apoptosis.

### The Loss of ULBP2 Results from Shedding

**Figure 4:**
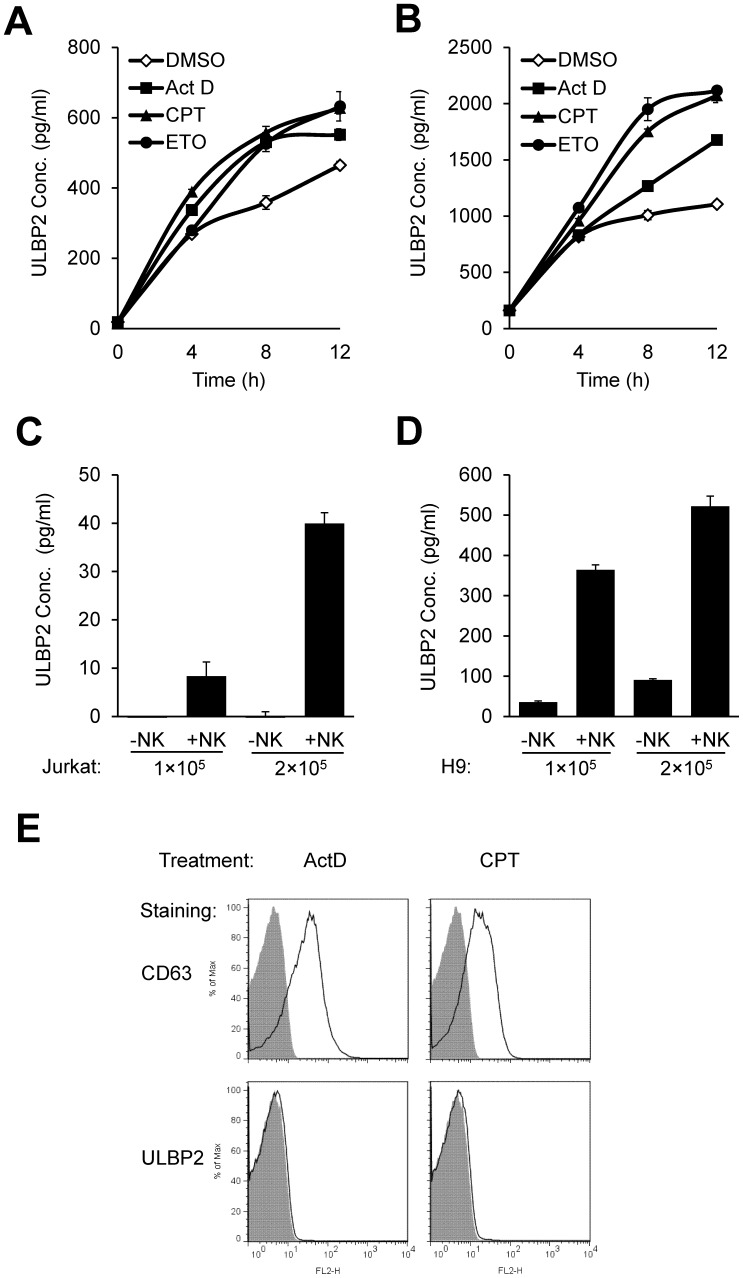
The spontaneous and NK cell-mediated shedding of ULBP2 from tumor cells. (A, B) Apoptosis-induced shedding of ULBP2 is more intense than spontaneous shedding of it. (A) 2.5×10^6^ Jurkat cells were treated with 2 µg/ml ActD, 4 µM CPT or 20 µM ETO for the indicated time in 0.5 ml serum-free RPMI 1640 medium. (B) 5×10^5^ cells H9 were treated with 2 µg/ml Act D, 4 µM CPT or 50 µM ETO for the indicated time in 0.5 ml serum-free RPMI 1640 medium. The supernatant was collected for ELISA assay. DMSO was used as a negative control. (C, D) NK cell-induced shedding of ULBP2. 1×10^5^ IL-2 expanded human NK cells were co-cultured with the indicated number of Jurkat (C) or H9 cells (D) for 2 hours, and then culture media were collected for ULBP2 ELISA. (E) Absence of ULBP2 in exosomes. 2×10^7^ H9 cells were resuspended in 10 ml serum-free RPMI 1640 medium and treated with ActD or CPT overnight. Exosome preparations from the resulting culture supernatants were used to coat 4-µm-diameter aldehyde/sulfate latex beads by passive adsorption. The coated beads were used for detection of CD63 and ULBP2 by flow cytometric analysis. Beads with exosomes coating are showed in solid lines, and beads without exosomes coating are showed in gray-shaded histograms as controls.

Notably, the surface reduction of ULBP2 is not associated with internalization, because cell surface expression of ULBP2 had not been changed when Jurkat cells were treated with Brefeldin A or Monensin for 4 hours in conventional cell culture, and both Brefeldin A and Monensin failed to block CPT-induced down-regulation of ULBP2 (Fig. S4). ULBP2 has been shown to shed from tumor cells and exist as a soluble form in sera of tumor patients [2], [3]. To test if the loss of ULBP2 from apoptotic cells resulted from shedding, soluble ULBP2 protein was measured in the supernatants of ActD-, CPT-, ETO- or DMSO-treated Jurkat cells by ELISA. The treatment with these apoptosis inducers led to more soluble ULBP2 production than the control DMSO treatment which reflects spontaneous shedding as previously reported by others (Fig. 4A). Similar results were also observed in apoptosis inducers-treated H9 cells (Fig. 4B). H9 cells expressed more cell surface ULBP2 than Jurkat cells, and therefore a higher concentration of ULBP2 was detected in H9 supernatants. Consistent with apoptotic compound treatments, NK cell-mediated cytotoxicity also induced shedding of ULBP2 from Jurkat (Fig. 4C) and H9 cells (Fig. 4D) into cell culture supernatants.

Since ELISA does not distinguish soluble proteins released by shedding from those presented in exosomes, we further used flow cytometry to investigate if soluble ULBP2 is associated with exosomal pathway. As shown in Fig. 4E, latex beads coated with exosomes prepared from ActD or CPT treated H9 cells were positive of exosome marker CD63 [18]. These exosome-coated beads, however, failed to be stained by the ULBP2 antibody. Thus, ULBP2 released from apoptotic cells was not associated with exosome exocytosis. Together, these results showed that ULBP2 was shed from target cells in response to NK cell-mediated cytolysis or apoptosis.

### NK Cell-induced Tumor Cell ULBP2 Shedding Differs from that of Spontaneous Shedding

**Figure 5:**
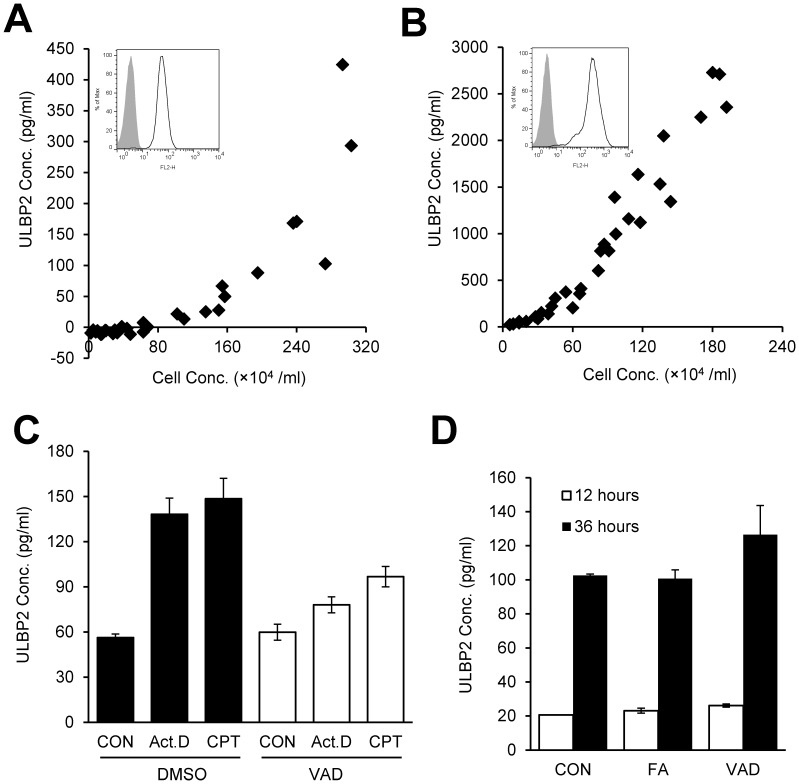
Spontaneous shedding of ULBP2 does not result from tumor cell apoptosis. (A, B) Spontaneous shedding of ULBP2 from Jurkat and H9 cells. Jurkat (A) or H9 (B) cells were cultured with initial seeding cell numbers ranging from 0.5×10^5^ to 4×10^5^ cells/ml in 48-well plates, the cells were harvested at various time points (from 17 to 98 hours) to achieve various cell densities, and their released ULBP2 in supernatants were determined by ELISA. The expression of ULBP2 was also been determined by FACS using PE-conjugated anti-human ULBP2 antibody. Cell surface expression of ULBP2 in Jurkat and H9 cells are shown in solid lines, and isotype controls are shown in gray-shaded histograms. (C) Shedding of ULBP2 from apoptotic cells. 4×10^6^ Jurkat cells were pre-treated with DMSO or 50 µM Z-VAD-FMK for 30 min, and then treated with ActD and CPT for 6 hours in serum free medium. The resulting culture supernatants were collected for ULBP2 ELISA. (D) Z-VAD-FMK fails to block spontaneous shedding of ULBP2. 8×10^4^ Jurkat cells were cultured in RPMI 1640 medium with 10% FBS in the presence of 50 µM Z-FA-FMK, Z-VAD-FMK or their carrier control DMSO for the indicated time. The culture supernatants were used to determine ULBP2 concentration.

To find out the key factor that controls spontaneous release of ULBP2 from tumor cells, we set up cell culture experiments to determine the relationship among culture time, seeding cell density, final cell density and concentration of ULBP2 in culture supernatants. Two ULBP2-expressing tumor cell lines, Jurkat and H9 cells, were cultured to achieve various cell densities by manipulating seeding cell number and/or culture time, and their released ULBP2 in supernatants were determined by ELISA. As shown in Fig. 5A and B, the concentration of released ULBP2 is in proportion to the final densities of cultured Jurkat and H9 cells. Interestingly, no matter how many cells were started with or how long the cells were cultured, once they achieved the same density, they tended to release the same amount of soluble ULBP2. In addition, H9 cells released approximately 10-fold more ULBP2 than Jurkat cells, which related to cell surface expression of ULBP2 on these two cell lines (Fig. 5A and B, inside histograms). These results are consistent with a previous publication which reported that the concentration of soluble ULBP2 has been identified as a reliable marker for tumor load [10].

Compared with spontaneous shedding as evident in the DMSO treatment or absence of NK cells, apoptotic compounds and NK cell-induced shedding lead to a higher rate of release of ULBP2 (Fig. 4). It is possible that spontaneous ULBP2 shedding resulted from a few apoptotic cells in normal cell culture. To further investigate the mechanism of spontaneous ULBP2 shedding, Jurkat cells were cultured in the presence of caspase inhibitor Z-VAD-FMK or its controls. While Z-VAD-FMK blocked ActD- and CPT-induced shedding of ULBP2, it did not affect the spontaneous ULBP2 shedding (Fig. 5C and D). These results demonstrate that spontaneous shedding of ULBP2 is not a result of apoptosis of tumor cells, and that the mechanism of apoptosis-induced shedding is different from that of spontaneous ULBP2 shedding.

### NK Cell-induced ULBP2 Shedding is Mediated by Metalloproteinase

**Figure 6:**
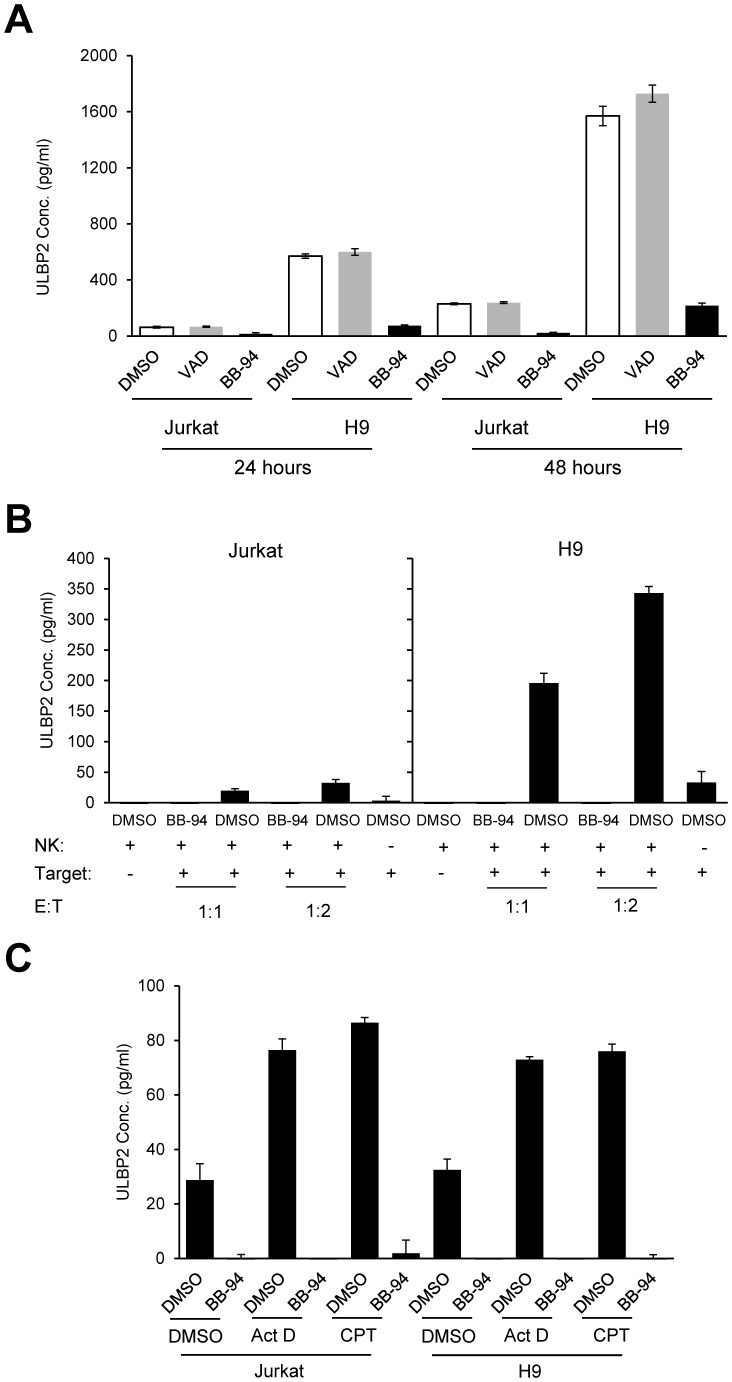
BB-94 abrogates NK cell-induced shedding of ULBP2 from apoptotic cells. (A) BB-94 blocks spontaneous shedding of ULBP2 from Jurkat and H9 cells. 10^6^ Jurkat cells or 5×10^5^ H9 cells were cultured in the presence of DMSO, Z-VAD-FMK and BB-94 for 24 or 48 hours in RPMI-1640 medium with 10% FBS, the resulting cell culture supernatants were collected for ELISA. (B) BB-94 abrogates NK cell-mediated shedding of ULBP2. 2×10^5^ Jurkat or H9 cells were incubated with IL-2 expanded primary human NK cells at the indicated E:T ratios for 4 hours, and the resulting cell culture supernatants were collected for ELISA. (C) BB-94 abrogates apoptotic compound-induced shedding of ULBP2. 4×10^6^ Jurkat cells or 4×10^5^ H9 cells were treated with ActD or CPT for 6 hours in serum-free RPMI 1640 medium, the culture supernatants were collected to measure ULBP2 concentration by ELISA.

**Figure 7:**
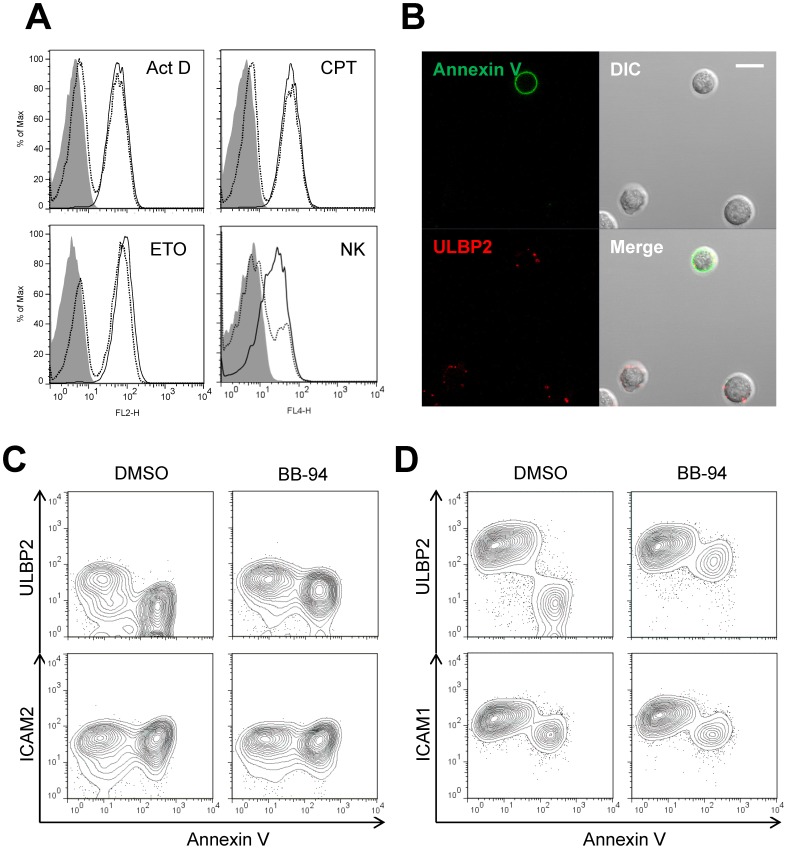
BB-94 rescues cell surface expression of ULBP2 in apoptotic cells. (A, B) BB-94 abrogates apoptotic compound-induced shedding of ULBP2. (A) Jurkat cells were treated with 2 µg/ml Act D, 4 µM CPT or 20 µM ETO for 4 hours in the presence or absence of BB-94. The treated cells were stained by PE-conjugated mouse anti-human ULBP2 antibodies, and then analyzed by flow cytometry. ULBP2 expression on control cells and BB-94 treated cells are shown in dotted lines and solid lines, respectively. PE-conjugated mouse IgG2a was used as an isotype control (gray-shaded). (B) Jurkat cells were treated with ActD for 2 hours in the presence of BB-94, and then ULBP2 and Annexin V staining was visualized by confocal microscopy. Scale bar, 10 µm. (C, D) BB-94 abrogates NK cell-mediated shedding of ULBP2. 10^5^ Jurkat (B) or H9 cells (C) were incubated with (+NK) or without (−NK) equal number of IL-2 expanded peripheral blood NK cells at 37°C for 2 hours. The resulting cell mixtures were stained by anti-human ULBP2 or ICAM1/2 antibodies, followed by APC-conjugated goat anti-mouse IgG antibody and Annexin V-PE staining, and then analyzed by flow cytometry. NK cells were excluded by FITC conjugated anti-human CD56 mAb staining.

Matrix metalloproteinases and members of ADAM family proteins are involved in spontaneous shedding of NKG2D ligands [19]–[21]. BB-94, also known as Batimastat, is a broad-spectrum metalloproteinase inhibitor that inhibits the matrix metalloproteinases and some ADAM family metalloproteinases such as Tumor necrosis factor-alpha cleaving enzyme [22]. As expected, BB-94 potently inhibited spontaneous shedding of ULBP2 from Jurkat and H9 cells during 24- or 48-hour culture in RPMI 1640 medium with 10% FBS, compared with its control DMSO and Z-VAD-FMK (Fig. 6A). To investigate if NK cell-induced apoptotic shedding of ULBP2 also required metalloproteinases, Jurkat and H9 cells were either incubated with NK cells (Fig. 6B) or treated with ActD or CPT (Fig. 6C), and then the culture supernatants were collected for detecting shedding of ULBP2. Both NK cell- and apoptotic compound-induced shedding of ULBP2 were abrogated by BB-94. Moreover, the metalloproteinase inhibitor BB-94 also rescued the cell surface expression of ULBP2 in ActD-, CPT- and ETO-induced apoptotic Jurkat cells (Fig. 7A). Furthermore, the expression of ULBP2 on apoptotic Jurkat cells was readily detectable by confocal microscopy in the presence of BB-94 (Fig. 7B). Similarly, NK cell-mediated loss of ULBP2 in Jurkat and H9 cells was inhibited by BB-94 (Fig. 7C and D). These data suggest that NK cell-mediated cytolysis-induced shedding of ULBP2 is also mediated by metalloproteinases.

### Effect of ULBP2 Shedding on NK Cell Effector Functions

**Figure 8:**
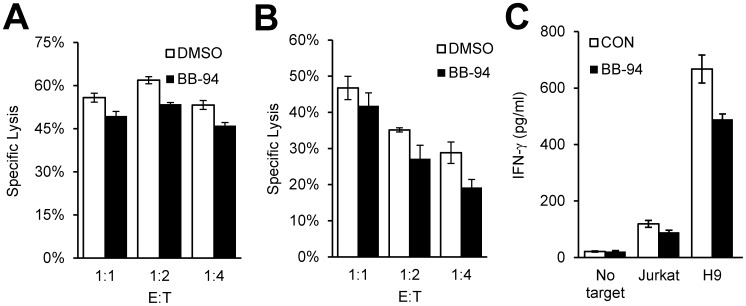
The role of shedding of ULBP2 in NK cell-mediated effector functions. (A, B) NK cell-mediated cytolysis in the presence of BB-94. Jurkat (A) and H9 (B) cells were used as target cells for cytotoxicity assay with IL-2 expanded primary human NK cells at the indicated E:T ratios. (C) The presence of BB-94 reduced IFN-γ production by NK cells. 10^5^ the indicated target cells were incubated with equal number of IL-2 expanded peripheral blood NK cells at 37°C for 6 hours. The concentration of IFN-γ in supernatants was determined by ELISA.

While NK cell-mediated cytolysis actively induced NKG2D ligand shedding on target cells, it is not clear whether such apoptotic shedding impacts NK cell effector functions. It is generally believed that once NK cells degranulate against a target cell, the apoptotic “dying” target cell becomes irrelevant to NK cell functions. To address if the ligand shedding affects NK cell functions, we investigated the effect of BB-94 on NK cell-mediated target cell lysis. While briefly inhibiting the shedding has no effect on the ULBP2 expression in live target cells, BB-94 prevented the loss of ULBP2 on annexin-V positive target cells (Fig. 7). Furthermore, the inhibition of shedding resulted in significant decreases in both NK cell-mediated cytolysis of target cells and IFN-γ production (Fig. 8A, B and C). The reduced NK cell effector functions may be due to non-productive NK cell engagement with apoptotic target cells when ULBP2 shedding is inhibited. Alternatively, ULBP2 shedding may facilitate NK cells release from target cells after degranulation. Either way, ligand shedding on apoptotic target cells enables NK cells to distinguish live versus dying target cells and to avoid potential non-productive engagement, which in turn preserves the effector functions.

## Discussion

Soluble NKG2D ligands are frequently found in leukemia patients [23]. In particular, ULBP2 has been identified as a predictive marker for cancer prognosis, and the levels of ULBP2 in sera from melanoma patients are strongly associated with tumor load [10]–[13]. As a result, better understanding the mechanism of shedding of ULBP2 may facilitate the development of ULBP2-based diagnosis methods for cancers. In this study, we showed that target cells lost their expression of ULBP2, but not ULBP1 and ULBP3, due to NK cell-mediated cytolysis as well as spontaneous shedding. NK cell-induced shedding of ULBP2 is dependent on apoptotic pathways and mediated by metalloproteinases. Like ULBP2, other NKG2D ligands, such as MICA/B, are also known to shed [21]. Moreover, due to the fact that NKG2D ligands primarily express on tumor or stressed cells, their rapid proteolytic shedding and, consequently, their increase in plasma concentration may be used to measure drug-induced tumor cell death and thus the efficacy of anti-tumor drugs.

While previous efforts primarily focused on tumor spontaneous release of NKG2D ligands and thus evading NK cell tumor surveillance, the influence of NK cells on NKG2D ligand shedding is much less defined. The cell surface expression of ULBP2 represents a net balance between gene synthesis and degradation, or shedding in this case. Therefore, the reduced ULBP2 surface expression on the tumor target in the presence of NK cells suggests a greater rate of shedding than that of spontaneous release. Indeed, the amount of released soluble ULBP2 and, hence, rate of ULBP2 shedding was much higher in the presence of NK cells than that of spontaneous shedding. Moreover, NK cell-induced shedding of ULBP2 is less dependent on target cell concentrations than the spontaneous ULBP2 shedding. In contrast to NK cell-induced shedding of ULBP2 on apoptotic cells, the spontaneous shedding of ULBP2, albeit at a lower level, occurs on non-apoptotic tumor cells. It is likely that the concentration of ULBP2 from tumor patients results from both spontaneous and apoptosis-induced shedding. Spontaneous shedding of NKG2D ligands is likely due to high expressions of metalloproteinases on tumor cells [19]–[21]. Moreover, ADAM17 has also been shown to contribute to shedding of MICB [20]. Since both spontaneous and NK cell/apoptosis-induced shedding of ULBP2 require metalloproteinases, it is likely that these two different shedding processes rely on the same metalloproteinases.

Several membrane proteins, such as L-selectin, Tumor-necrosis factor receptor-1 and Interleukin 6 receptor, are known to be released through apoptosis-induced shedding [24]–[26], and we also observed the shedding of L-selectin (CD62L) from Jurkat cells during NK cell-mediated cytolysis (Fig. S5). The apoptosis induced-shedding may be related to an increase in processed metalloproteinase during apoptosis [26]. However, in this study, both Brefeldin A and Monensin failed to block shedding of ULBP2, which are known to abrogate endoplasmic reticulum-to-Golgi and trans-Golgi transportation and therefore block extracellular protein expression, indicating that newly synthesized or processed metalloproteinases may not play a role in ULBP2 shedding. As a result, the shedding of ULBP2 may depend on activated metalloproteinases existing on the cell surface, which may get the chance to approach their substrates during apoptosis. Consistent with this, Latrunculin B and Cytochalasin D which disrupt actin microfilaments and destabilize plasma membrane structure were able to partially inhibit shedding of ULBP2 (Fig. S4B).

Abnormality of NK cells has long been observed in patients with autoimmune diseases, and the disruption of NK cell tolerance by overexpression of stress-induced ligands for activating receptors is believed to induce tissue damage [27]–[29]. For example, overexpression of NKG2D ligands may contribute to pathogenesis of Celiac disease, Crohn's disease, Type I diabetes, Behcet's disease and Alopecia areata [27]–[29]. Consequently, the ability to specifically regulate NK cell effector functions through inhibiting NKG2D ligand shedding by metalloproteinases or apoptosis inhibitors may present potential therapeutic benefit by preventing or alleviating pathogenesis in certain autoimmune diseases.

## Supporting Information

Figure S1 **NK cell-mediated loss of ULBP2.** Jurkat cells were incubated with 10^5^ IL-2 expanded peripheral blood NK cells at the indicated E:T ratios at 37°C for 2 hours. The resulting cell mixtures were stained by PE-conjugated mouse anti-human ULBP1 or ULBP2 antibodies and analyzed by flow cytometry (solid lines). NK and target cells were distinguished by APC-conjugated anti-human CD56 mAb staining. Isotype controls are shown in gray-shaded histograms.
(TIF)

Figure S2 **Apoptotic compound treatment doesn’t affect cell surface expression of ULBP1, CD95 and HLA class I.** Jurkat cells were treated with 4 µg/ml ActD, 4 µM CPT, 25 µM ETO or DMSO for 4 hours in serum-free RPMI 1640 medium, and then were collected for flow cytometry staining. Mouse anti-human ULBP1, CD95 and HLA-ABC antibodies were used. The expression of ULBP1, CD95 and HLA-ABC on DMSO-treated control cells and apoptotic compound-treated cells are shown in dotted lines and solid lines, respectively. Isotype controls are shown in gray-shaded histograms.
(TIF)

Figure S3 **Heat shock-induced apoptosis leads to down-regulation of cell surface ULBP2 in Jurkat cells.** (A, B) Jurkat cells were heated at 45°C for 30 min, and then incubated on ice (CON) or at 37°C for another 2 hours (Heat Shock). The treated cells were stained by biotin-labeled goat anti-human ULBP1 (A) or ULBP2 (B) polyclonal antibodies, followed by APC-conjugated streptavidin and Annexin V-FITC staining, and then analyzed by flow cytometry.
(TIF)

Figure S4 **Effect of Brefeldin A, Monensin, Latrunculin B and Cytochalasin D on loss of ULBP2.** (A) Jurkat cells were treated with Brefeldin A (BFA) or Monensin (MON) for 4 hours in the presence or absence of CPT in serum-free RPMI 1640 medium, and then were collected for flow cytometric staining. PE-conjugated mouse anti-human ULBP2 antibodies were used. ULBP2 expression on control cells and treated cells are shown in dotted lines and solid lines, respectively. The expression of ULBP2 on CPT alone treated cells (without BFA/MON) are shown in dashed lines. PE-conjugated mouse IgG2a was used as an isotype control (gray-shaded). (B) Latrunculin B and Cytochalasin D inhibit shedding of ULBP2. Jurkat cells were treated with Act D and CPT for 4 hours in the presence of Latrunculin B or Cytochalasin D in serum-free RPMI 1640 medium, and then were collected for flow cytometric staining. PE-conjugated mouse anti-human ULBP2 antibodies were used. ULBP2 expression on control cells (with ActD or CPT treatment) and Latrunculin B or Cytochalasin D treated cells are shown in dotted lines and solid lines, respectively. PE-conjugated mouse IgG2a was used as an isotype control (gray-shaded).
(TIF)

Figure S5 **NK cell-mediated loss of L-selectin and ULBP2.** 10^5^ Jurkat were incubated with (+NK) or without (−NK) in an equal number of IL-2 expanded peripheral blood NK cells at 37°C for 2 hours. The resulting cell mixtures were stained by PE-conjugated anti-human L-selectin (CD62L) or ULBP2 antibodies, followed by Annexin V-FITC staining, and then analyzed by flow cytometry. NK cells were excluded by APC conjugated anti-human CD56 mAb staining.
(TIF)
