# Supplementary figures and images for: Natural Killer Cell-Mediated Shedding of ULBP2
Source: PLoS One. 2014 Mar 10;9(3):e91133. doi: 10.1371/journal.pone.0091133 (PMC3948742; doi:10.1371/journal.pone.0091133)

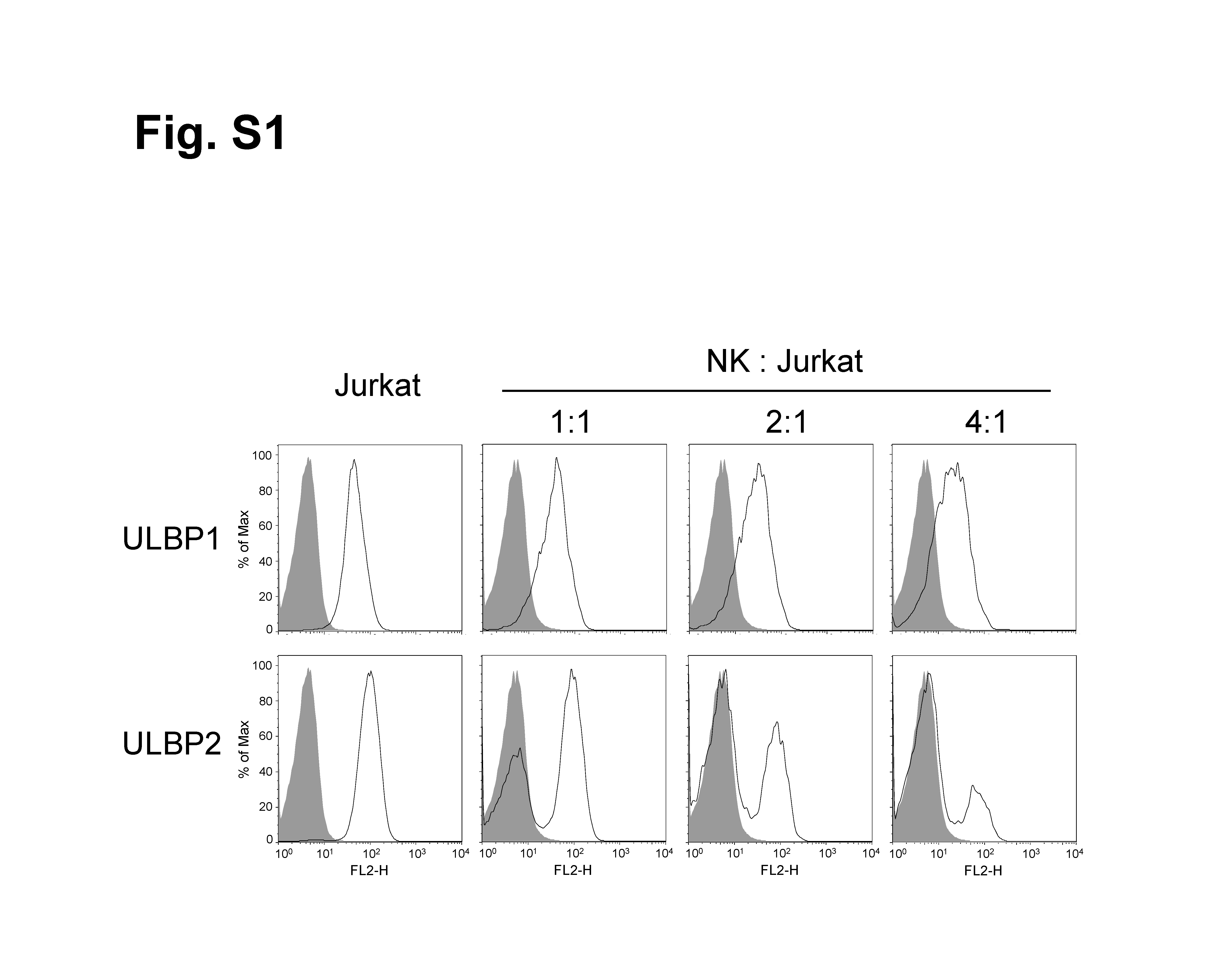

Supplement: Figure S1 — NK cell-mediated loss of ULBP2. Jurkat cells were incubated with 105 IL-2 expanded peripheral blood NK cells at the indicated E:T ratios at 37°C for 2 hours. The resulting cell mixtures were stained by PE-conjugated mouse anti-human ULBP1 or ULBP2 antibodies and analyzed by flow cytometry (solid lines). NK and target cells were distinguished by APC-conjugated anti-human CD56 mAb staining. Isotype controls are shown in gray-shaded histograms. (TIF) [file pone.0091133.s001.tif]

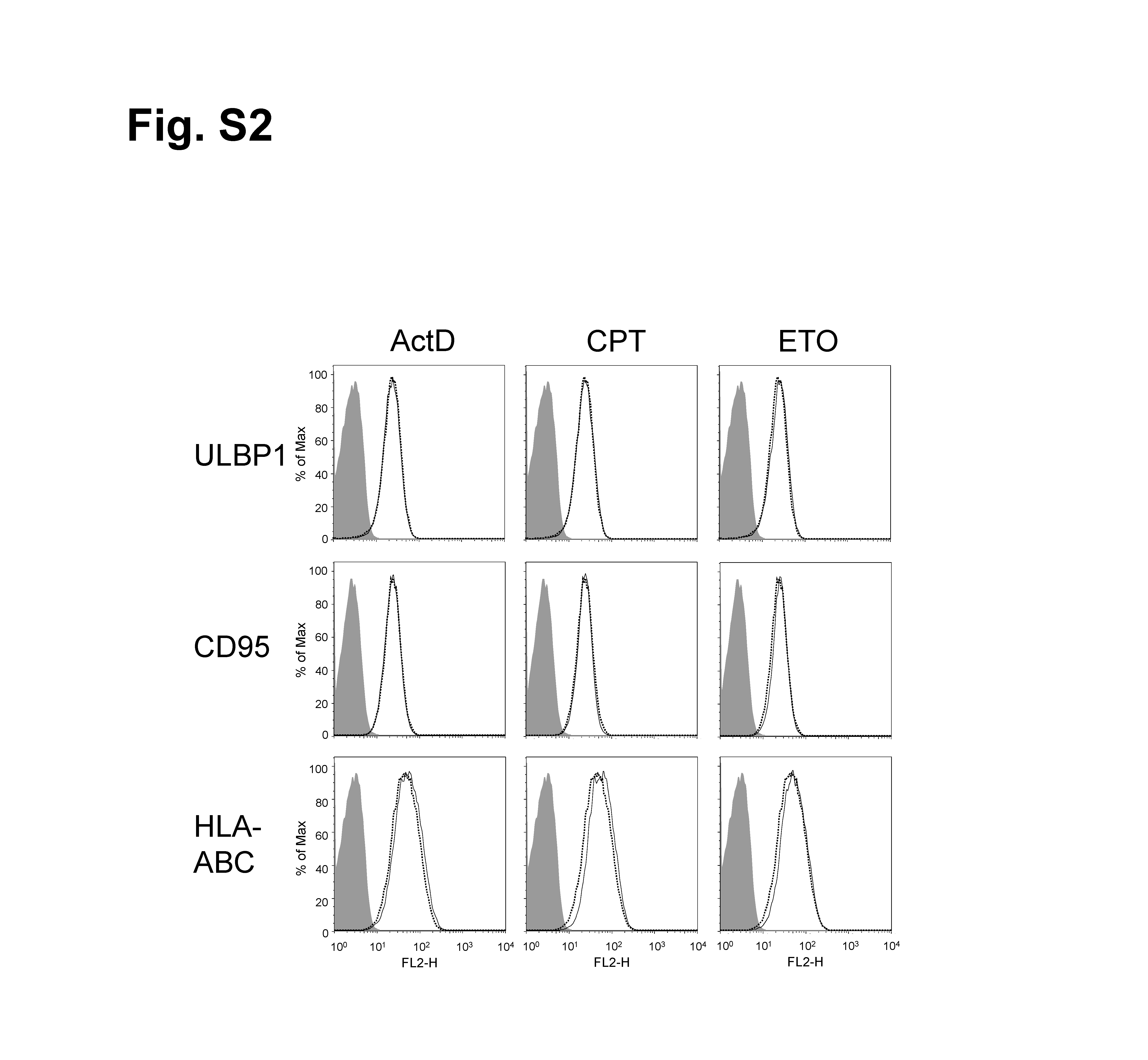

Supplement: Figure S2 — Apoptotic compound treatment doesn’t affect cell surface expression of ULBP1, CD95 and HLA class I. Jurkat cells were treated with 4 µg/ml ActD, 4 µM CPT, 25 µM ETO or DMSO for 4 hours in serum-free RPMI 1640 medium, and then were collected for flow cytometry staining. Mouse anti-human ULBP1, CD95 and HLA-ABC antibodies were used. The expression of ULBP1, CD95 and HLA-ABC on DMSO-treated control cells and apoptotic compound-treated cells are shown in dotted lines and solid lines, respectively. Isotype controls are shown in gray-shaded histograms. (TIF) [file pone.0091133.s002.tif]

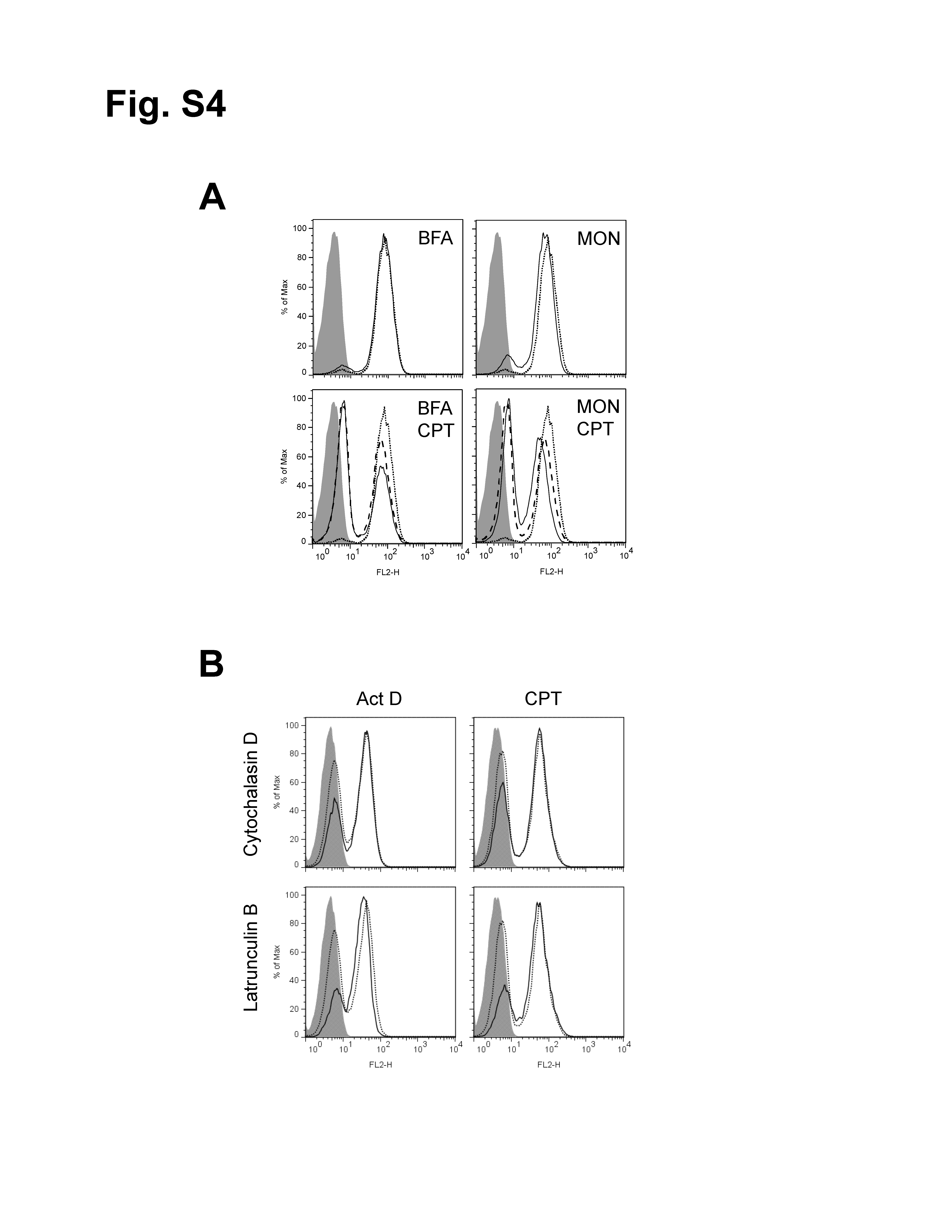

Supplement: Figure S4 — Effect of Brefeldin A, Monensin, Latrunculin B and Cytochalasin D on loss of ULBP2. (A) Jurkat cells were treated with Brefeldin A (BFA) or Monensin (MON) for 4 hours in the presence or absence of CPT in serum-free RPMI 1640 medium, and then were collected for flow cytometric staining. PE-conjugated mouse anti-human ULBP2 antibodies were used. ULBP2 expression on control cells and treated cells are shown in dotted lines and solid lines, respectively. The expression of ULBP2 on CPT alone treated cells (without BFA/MON) are shown in dashed lines. PE-conjugated mouse IgG2a was used as an isotype control (gray-shaded). (B) Latrunculin B and Cytochalasin D inhibit shedding of ULBP2. Jurkat cells were treated with Act D and CPT for 4 hours in the presence of Latrunculin B or Cytochalasin D in serum-free RPMI 1640 medium, and then were collected for flow cytometric staining. PE-conjugated mouse anti-human ULBP2 antibodies were used. ULBP2 expression on control cells (with ActD or CPT treatment) and Latrunculin B or Cytochalasin D treated cells are shown in dotted lines and solid lines, respectively. PE-conjugated mouse IgG2a was used as an isotype control (gray-shaded). (TIF) [file pone.0091133.s004.tif]
